# Why Emergency Physicians Should Care About the Salton Sea

**DOI:** 10.5811/westjem.2017.8.36034

**Published:** 2017-09-21

**Authors:** John R. Marshall

**Affiliations:** University of California, Irvine, Department of Emergency Medicine, Irvine, California

The deterioration of the ecosystem surrounding the Salton Sea, a 376-square mile lake located between Imperial and Riverside Counties in Southern California^1^ has an important effect on the health of the surrounding impoverished population. The greater Salton Sea area comprises 177,000 people demographically divided into 80.9% Latino, 13.7% White/Non-Hispanic, and 10.5% African American.^2^ Median income and poverty percentiles in the area are 23.02% and $35,000 compared to the rest of California at 15.28% and $61,400 respectively.^3^,^4^ This area is also disproportionately affected by pediatric asthma with 20% of its pediatric population diagnosed with asthma compared to 8% nationally.^5^ Currently, emergency departments in Imperial County treat three times more pediatric asthma visits than elsewhere in California.^5^,^6^ Recently, there has been new governmental, academic, and community interest in this issue, and as emergency physicians we have a unique opportunity to become involved in the health of the Salton Sea as well as the surrounding community.

The Salton Sea is a terminal body of water without outflow and relies on agricultural runoff, and the New, Whitewater, and Alamo rivers to maintain its water level. The last filling of the lake occurred in 1904 when the Colorado River flooded, and the surrounding area has since become heavily populated and a vital part of the Coachella Valley economy and integral part of the Southern California ecosystem.^7^ Over the last decade, the lake has been decreasing in size due to evaporation, increasing its salinity. Without efforts to sustain the sea, it is projected to decrease to 1/3 its size in the next 50 years, potentially crippling the local economy, with catastrophic effects on the health of the impoverished surrounding population, and adversely affecting air quality in Southern California.^8^

Approximately 1,300,000 acre-feet of water each year evaporate from the Salton Sea (one acre-foot = 326,000 gallons).^1^ Without outflow, it is dependent on river water diversion as well as runoff from agricultural irrigation to equal its evaporative rate. In recent years, water levels have dropped drastically due to the 2003 Quantification Settlement Agreement, which diverted large amounts of river water to urban San Diego.^9^ This uncompensated evaporation causes salinity levels of the lake to increase. If there is no intervention to address these rising salinity levels, life in the lake will become unsustainable, crippling the fishing, agricultural and recreation economies, as well as having a drastic effect on the ecosystem. 1,7–9 Greater exposed lakebed from evaporation will create dust storms, increasing the particulate burden in the atmosphere as well as releasing toxic chemicals such as hydrogen sulfide, arsenic, and pesticides from irrigation run off.^10^ Currently, the particulate matter in the atmosphere surrounding the lake exceeds California and National Ambient Air quality standards, but it is currently unclear how much of this is attributable to the lake evaporation.^11^ These pollutants act as a pulmonary irritant and likely play a role in the high incidence of chronic lung disease in the area.^10^,^11^ Additionally, these dust particles are very fine and may spread, affecting the air quality of the Los Angeles and San Diego metropolitan areas.^1^

Though certain options like desalination and water diversion are possible to restore lake volume and improve salinity, they are very costly and water diversion is difficult in the drought-ridden Southwest. Fortunately, there are other strategies to restore and protect the Salton Sea. Potential renewable energy projects endorsed by the Geothermal Energy Association to take place at or around the sea (i.e. solar, geothermal, wind), have the potential to finance Salton Sea air quality and habitat restoration.^12^ Additionally, surrounding areas have become booming residential areas due to the lower cost of land. Between 2005–2012 the population grew by 10.5% and is anticipated to double in 50 years.^13^ The projected growth is a potential strategic opportunity to mobilize people fleeing high Los Angeles metro-area housing prices to take interest in the restoration and preservation of the Salton Sea.

Recently there has been increased governmental interest in the health of the Salton Sea. Senate Bill 615 (SB-615) requires the California Natural Resources Agency to develop and fund a 10-year plan by 2018 with the U.S. Department of the Interior to address and improve habitat and air quality and projected lakebed exposure of the Salton Sea. This will be done in consultation with the Salton Sea Authority, a nonprofit agency dedicated to its revitalization.^14^ This bill, which was passed in June of 2017, contains a series of declarations regarding the deteriorating physical condition of the Salton Sea, the importance of habitat restoration projects, and the importance of efforts to control dust emissions improving air quality in the area.^14^

Current research by the University of Southern California and the University of Iowa on lakebed composition, as well as airborne particulate matter, aims to establish the composition of the lakebed and released air particles, as well as community-level research to establish the incidence of pulmonary disease in the area. This is key to determining particle toxicity and the extent to which evaporation affects the atmosphere as well as how it will affect the current population.^15^

The revitalization of the Salton Sea and improving the air quality of the area will require a multi-tiered effort of local community empowerment, local and state government, healthcare providers, researchers and environmental activists. This is not only pertinent to the greater Salton Sea area, but may reach those in adjacent, heavily populated metropolitan areas. It is our duty as emergency physicians to help those suffering from health disparities through participation in research, community empowerment, and involvement with local government.
